# Nasal Delivery of a Commensal *Pasteurellaceae* Species Inhibits Nontypeable Haemophilus influenzae Colonization and Delays Onset of Otitis Media in Mice

**DOI:** 10.1128/IAI.00685-19

**Published:** 2020-03-23

**Authors:** Caitlyn M. Granland, Naomi M. Scott, Jean-Francois Lauzon-Joset, Jeroen D. Langereis, Camilla de Gier, Katrien M. J. Sutherland, Sharon L. Clark, Janessa L. Pickering, Ruth B. Thornton, Peter C. Richmond, Deborah H. Strickland, Lea-Ann S. Kirkham

**Affiliations:** a Wesfarmers Centre of Vaccines and Infectious Diseases, Telethon Kids Institute, Perth, Australia; b Section Pediatric Infectious Diseases, Laboratory of Medical Immunology, Radboud Institute for Molecular Life Sciences, Radboudumc, Nijmegen, The Netherlands; c Radboud Center for Infectious Diseases, Radboudumc, Nijmegen, The Netherlands; d Division of Paediatrics, Faculty of Medicine, University of Western Australia, Perth, Australia; e School of Biomedical Sciences, Faculty of Health and Medical Sciences, University of Western Australia, Perth, Australia; f School of Life Sciences, College of Medical, Veterinary and Life Sciences, University of Glasgow, Glasgow, United Kingdom; g Centre for Child Health Research, University of Western Australia, Perth, Australia; University of Pennsylvania

**Keywords:** *Haemophilus influenzae*, *Muribacter muris*, bacterial therapy, microbial interference, otitis media

## Abstract

Nasopharyngeal colonization with nontypeable Haemophilus influenzae (NTHi) is a prerequisite for developing NTHi-associated infections, including otitis media. Therapies that block NTHi colonization may prevent disease development. We previously demonstrated that Haemophilus haemolyticus, a closely related human commensal, can inhibit NTHi colonization and infection of human respiratory epithelium *in vitro*. We have now assessed whether Muribacter muris (a rodent commensal from the same family) can prevent NTHi colonization and disease *in vivo* using a murine NTHi otitis media model. Otitis media was modeled in BALB/c mice using coinfection with 1 × 10^4.5^ PFU of influenza A virus MEM H3N2, followed by intranasal challenge with 5 × 10^7^ CFU of NTHi R2866 Spec^r^. Mice were pretreated or not with an intranasal inoculation of 5 × 10^7^ CFU *M. muris* 24 h before coinfection. NTHi and *M. muris* viable counts and inflammatory mediators (gamma interferon [IFN-γ], interleukin-1β [IL-1β], IL-6, keratinocyte chemoattractant [KC], and IL-10) were measured in nasal washes and middle ear tissue homogenate. *M. muris* pretreatment decreased the median colonization density of NTHi from 6 × 10^5^ CFU/ml to 9 × 10^3^ CFU/ml (*P* = 0.0004). Only 1/12 *M. muris*-pretreated mice developed otitis media on day 5 compared to 8/15 mice with no pretreatment (8% versus 53%, *P* = 0.0192). Inflammation, clinical score, and weight loss were also lower in *M. muris*-pretreated mice. We have demonstrated that a single dose of a closely related commensal can delay onset of NTHi otitis media *in vivo*. Human challenge studies investigating prevention of NTHi colonization are warranted to reduce the global burden of otitis media and other NTHi diseases.

## Abstract (precis)

Nasopharyngeal colonization with nontypeable Haemophilus influenzae (NTHi) is a prerequisite for developing NTHi-associated infections, including otitis media. Therapies that block NTHi colonization may prevent disease development. We previously demonstrated that Haemophilus haemolyticus, a closely related human commensal, can inhibit NTHi colonization and infection of human respiratory epithelium *in vitro*. We have now assessed whether Muribacter muris (a rodent commensal from the same family) can prevent NTHi colonization and disease *in vivo* using a murine NTHi otitis media model.

## INTRODUCTION

Nontypeable Haemophilus influenzae (NTHi) is an opportunistic pathogen that colonizes the upper respiratory tract of humans. NTHi belongs to the *Pasteurellaceae* family, consisting of a diverse group of Gram-negative facultative anaerobic bacteria. Many members of the family reside on mucosal surfaces of the respiratory tract of mammals and birds. Of the *Haemophilus* species, nine exhibit host specificity for humans (1), including H. influenzae and the closely related respiratory tract commensal Haemophilus haemolyticus (2, 3). Asymptomatic nasopharyngeal carriage of NTHi is common, especially in pediatric populations (4). NTHi can also cause a range of respiratory-related diseases, including middle ear infections (otitis media), sinusitis, conjunctivitis, and pharyngitis, as well as acute exacerbations in patients with chronic lung diseases and invasive diseases, such as meningitis and bacteremia (4, 5). There are currently no licensed preventative therapies that specifically target NTHi colonization and/or disease (6).

The burden of NTHi-associated otitis media is high. Studies from North America, Europe, and Oceania have shown that NTHi accounts for 45 to 61% of all recorded otitis media cases (7–10), and a systematic review of otitis media etiology from 1970 to 2014 found NTHi to be the predominant otopathogen (11). With over 700 million annual cases of acute otitis media throughout the world, ∼31 million chronic infections, and ∼21,000 deaths from otitis media complications every year (12), preventing NTHi otitis media would have a significant impact on reducing global morbidity. In addition, prevention of NTHi otitis media would have a significant impact on reducing antibiotic use, with otitis media being the main reason for antibiotic prescriptions in children (13).

Colonization of the nasopharynx with NTHi is a prerequisite for developing and transmitting disease, with early life colonization and increased NTHi density in the nasopharynx associated with the onset of otitis media and other respiratory infections (14–17). Thus, preventing or eradicating NTHi colonization of the nasopharynx is an attractive target to stop progression to disease and minimize host-to-host transmission.

Microbial interference offers a potential solution for inhibiting NTHi colonization and preventing development of disease. This approach involves the use of commensal bacteria to compete with pathobionts for binding sites, nutrients, and space in order to beneficially alter the host microflora. Microbial interference is currently being investigated to combat a range of bacterial infections, including pneumococcal otitis media in children using a nasal alpha-hemolytic streptococcal probiotic spray (18), experimental meningococcal meningitis in mice using intranasal delivery of the closely related commensal Neisseria lactamica (19), and pneumococcal pneumonia in mice using intranasal delivery of Streptococcus mitis (20). From these and other studies (21, 22), it appears that microbial interference requires the commensal and pathogenic species to belong to the same family of bacteria and be able to colonize the same niche.

We have previously demonstrated that the human respiratory tract commensal H. haemolyticus can be used to prevent NTHi infection of epithelial cells *in vitro* (23), reducing both NTHi attachment and invasion, indicating that microbial interference may occur between these two species. Some H. haemolyticus isolates have been found to produce a bacteriocin-like substance that specifically inhibits NTHi growth (24), further supporting a role for H. haemolyticus as a bacterial therapy to prevent NTHi disease. H. haemolyticus does not colonize mice (our unpublished data); therefore, we sought alternatives to further investigate microbial interference of NTHi *in vivo*. In this study, we have used Muribacter muris, a closely related rodent equivalent of H. haemolyticus from the *Pasteurellaceae* family (25), in a murine model of NTHi acute otitis media. We have assessed whether intranasal pretreatment of mice with *M. muris* can be used to prevent NTHi colonization and development of disease.

## RESULTS

### Intranasal treatment of mice with *M. muris* can temporarily reduce NTHi colonization and prevent development of NTHi otitis media.

**FIG 1:**
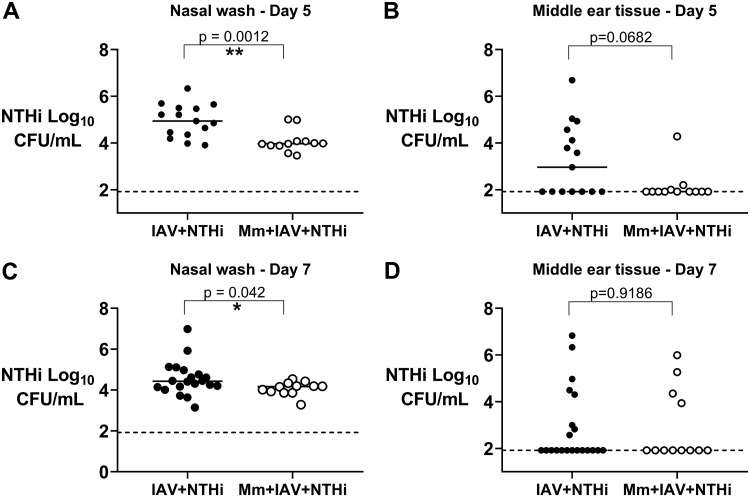
Pretreatment with *M. muris* reduces NTHi colonization and prevents development of otitis media. Nontypeable Haemophilus influenzae (NTHi) density in log_10_ CFU/ml from nasal washes and middle ear tissue homogenates of mice on day 5 (A and B) and day 7 (C and D) postchallenge. Each circle represents an individual mouse. Filled black circles represent mice that did not receive *M. muris* pretreatment prior to IAV + NTHi, open white circles represent mice that were pretreated intranasally with a single dose of 5 × 10^7^ CFU of *M. muris* on day −1 prior to IAV + NTHi (*M. muris* [Mm] + IAV + NTHi). Horizontal bars depict the median NTHi density, and the dashed line represents the limit of quantification. *, *P* < 0.05; **, *P* < 0.01; ***, *P* < 0.001.

Intranasal administration of 5 × 10^7^ CFU of *M. muris* to mice prior to challenge with influenza A/Memphis/1/71 H3N2 virus (IAV) and NTHi (*M. muris* + IAV + NTHi group) reduced the NTHi density recovered from the nose of mice on day 5 from a median log 4.94 CFU/ml (95% confidence interval [CI] of median 4.36 to 5.50) to a median log 3.97 (95% CI, 3.88 to 4.08) when compared with that of no pretreatment (IAV + NTHi group; *P* < 0.001) (Fig. 1A). *M. muris* pretreatment also prevented development of NTHi otitis media by day 5, with only 1 out of 12 (8%) *M. muris*-treated mice developing NTHi otitis media compared with 53% (8/15) of mice given no *M. muris* pretreatment (*P* = 0.019) (Fig. 1B). The median density of NTHi in the middle ear on day 5 reduced from median log 2.96 (95% CI, 1.92 to 4.56) to median log 1.92 (95% CI, 1.92 to 2.00) (*P* = 0.068). By day 7, the impact of *M. muris* pretreatment on NTHi colonization was less evident with a lower significant decrease in median NTHi density in nasal washes from *M. muris*-pretreated versus untreated mice (log 4.17 [95% CI, 3.87 to 4.34] versus log 4.43 [95% CI, 4.17 to 4.77]; *P* = 0.042) (Fig. 1C). There was no difference in the proportion of mice that had otitis media by day 7, with 4 out of 12 (33%) *M. muris*-treated mice developing NTHi otitis media compared to 8 out of 21 (38%) mice with no *M. muris* pretreatment (*P* > 0.999) (Fig. 1D). The median log density of NTHi recovered from the middle ear on day 7 was the same for each group at log 1.92 (*M. muris* treated = 1.92 [95% CI, 1.92 to 4.35] and untreated = 1.92 [95% CI, 1.92 to 3.00]; *P* = 0.981), which is at the limit of quantification.

### *M. muris* pretreatment reduces the nasal inflammatory response to IAV and NTHi.

**FIG 2:**
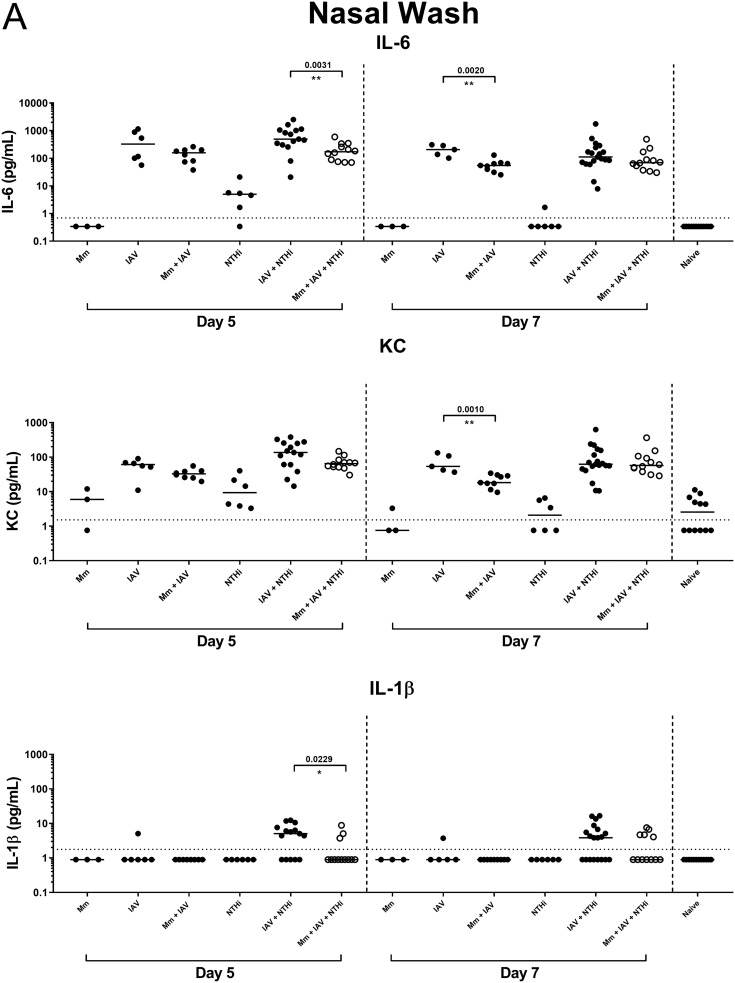

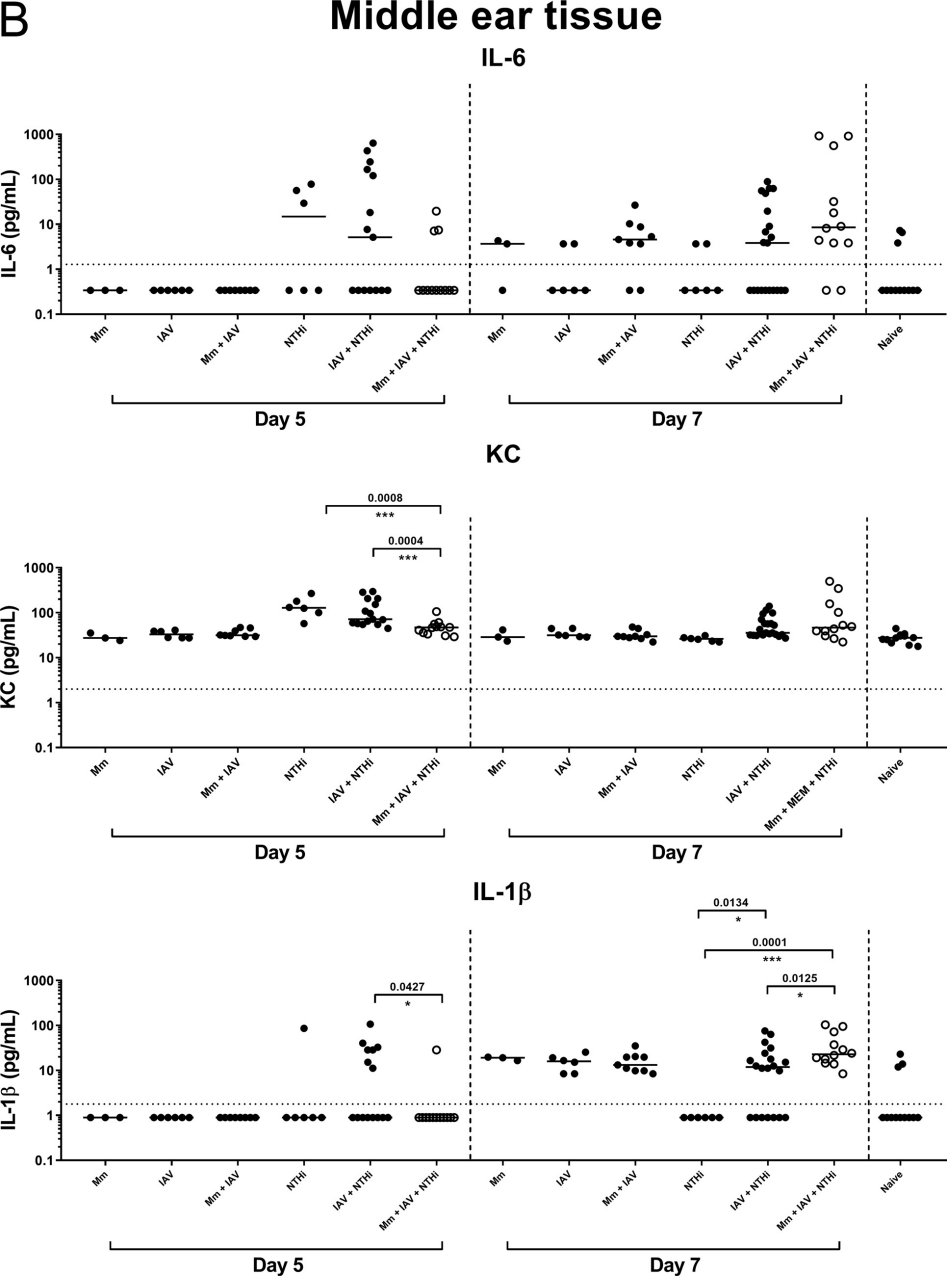
*M. muris* pretreatment reduces inflammation in the upper respiratory tract. Inflammatory mediator levels in nasal washes (A) and middle ear tissue homogenate (B) from mice on day 5 and day 7 postchallenge. Each circle represents an individual mouse. Filled black circles represent mice in control groups given *M. muris* only (Mm), influenza A virus only (IAV), NTHi only, or in combinations. The open white circles represent mice that were pretreated intranasally with a single dose of 5 × 10^7^ CFU of *M. muris* on day −1 prior to undergoing influenza virus and NTHi challenge (*M. muris* + IAV + NTHi). Horizontal bars depict the median analyte titer in picograms per milliliter, and the dashed line represents the assay limit of detection. *, *P* < 0.05; **, *P* < 0.01; ***, *P* < 0.001.

On day 5, the median titers of inflammatory mediators interleukin-6 (IL-6) and IL-1β were significantly lower in nasal washes from mice in the NTHi otitis media model that were pretreated with *M. muris* (*M. muris* + IAV + NTHi) than from mice with no *M. muris* pretreatment (IAV + NTHi) as follows: IL-6, 171 pg/ml (95% CI, 75.7 to 340) versus 492 pg/ml (95% CI, 304.8 to 1,055), *P* = 0.0031; IL-1β, 0.9 pg/ml (95% CI, 0.895 to 3.73) versus 5.1 pg/ml (95% CI, 0.895 to 7.61), *P* = 0.0229 (Fig. 2A). Keratinocyte chemoattractant (KC) levels were also lower (although not significantly) in nasal washes from the *M. muris* + IAV + NTHi group than from the IAV + NTHi group as follows: 65 pg/ml (95% CI, 51.07 to 83.62) versus 137 pg/ml (95% CI, 60.3 to 255.8), *P* = 0.0725 (Fig. 2A). By day 7, there was no significant difference between median inflammatory mediator titers from the *M. muris*-pretreated (*M. muris* + IAV + NTHi) versus untreated NTHi otitis media group (IAV + NTHi), though titers were higher than those in naive mice or in mice administered *M. muris* or NTHi alone (*P* < 0.05 for IL-6, KC, and IL-1β) (Fig. 2A). IAV administration alone increased the median IL-6 and KC levels in the nasal washes compared with those of the naive, *M. muris* only, or NTHi only treated mice on day 5 and day 7. Interestingly, mice that were pretreated with *M. muris* prior to IAV challenge (*M. muris* + IAV) had less inflammation in their nares than mice given IAV alone, and this was most pronounced on day 7 for both IL-6 and KC (*P* = 0.0020 and *P* = 0.0010, respectively) (Fig. 2A). Gamma interferon (IFN-γ) and IL-10 were not detected in nasal washes from any treatment.

### Intranasal pretreatment with *M. muris* temporarily reduces inflammatory mediator levels in the middle ear tissue of mice in the NTHi otitis media model.

On day 5, IL-6 was elevated in the middle ears of mice receiving NTHi, either alone or in combination with IAV (IAV + NTHi) and *M. muris* (*M. muris* + IAV + NTHi) (Fig. 2B). There was no difference between IL-6 levels in the ears of mice pretreated with *M. muris* (*M. muris* + IAV + NTHi) compared to untreated mice (IAV + NTHi). KC levels were also elevated in the ears of mice that received NTHi on day 5; however, mice that were pretreated with *M. muris* (*M. muris* + IAV + NTHi) had significantly reduced KC titers compared with those of mice that received NTHi challenge alone (*P* = 0.0008) or IAV + NTHi challenge (*P* = 0.0004) (Fig. 2B). By day 7, the KC titer returned to baseline value in all of the groups. Pretreatment of mice with *M. muris* (*M. muris* + IAV + NTHi) prevented the elevated IL-1β response observed in the ears of mice in the NTHi otitis media group (IAV + NTHi) on day 5 (*P* = 0.0427); however, this was reversed by day 7 where mice receiving *M. muris* pretreatment had higher median IL-1β titers in their ears than untreated mice in the otitis media model (*P* = 0.0125) (Fig. 2B). IFN-γ and IL-10 levels were either very low or not detected in the middle ear tissue, with no difference between median titers for any groups.

### Mice that were pretreated with *M. muris* had better clinical outcomes in the NTHi otitis media model.

**FIG 3:**
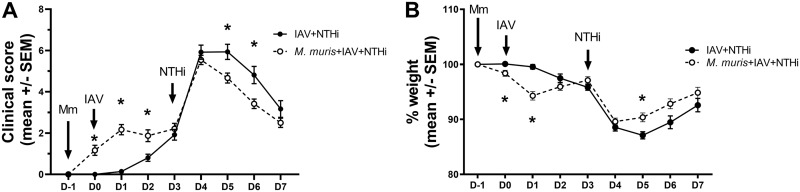
Pretreatment of mice with *M. muris* improves clinical outcomes and reduces otitis media-induced weight loss in the NTHi otitis media model. Mean clinical score (A) and weight loss (B) for mice with (closed black circles; *n* = 12 by day 7) or without (white circles; *n* = 15 by day 7) *M. muris* (Mm) pretreatment. Values are presented as the mean ± standard error of the mean. D, days postchallenge with influenza A virus challenge (IAV) as the reference point. NTHi was administered on day 3 and mice monitored to day 7; *, *P* < 0.01 when compared between treatment groups.

Administration of a single intranasal dose of *M. muris* reduced disease symptoms over the 7-day time frame in the NTHi otitis media model, with lower clinical scores (Fig. 3A) and less weight loss (Fig. 3B) than mice with no *M. muris* pretreatment (IAV + NTHi). On day 6, the clinical score of mice in the *M. muris*-pretreated group (*M. muris* + IAV + NTHi) was significantly lower than those that did not receive *M. muris* pretreatment (IAV + NTHi) (*P* < 0.05) (Fig. 3A). *M. muris* administration alone had no impact on the condition of the mice, with a mean clinical score of 0 to 1 over the 7 days (see Fig. S1A in the supplemental material). *M. muris* only treatment also had no significant impact on weight loss (see Fig. S1B). On the day after IAV challenge (day 1), the *M. muris*-treated group (*M. muris* + IAV + NTHi) lost 5% of body weight compared with that of the IAV-treated group (IAV + NTHi) (*P* < 0.01) (Fig. 3B). Similar weight loss also occurred following IAV challenge in the groups that did not receive *M. muris* pretreatment, but this was observed on day 2 after IAV challenge rather than on day 1 (see Fig. 3B for IAV + NTHi; see also Fig. S1B for IAV only and IAV + NTHi). However, upon NTHi challenge, the mice that were pretreated with *M. muris* (*M. muris* + IAV + NTHi) had significantly less weight loss than those that had no *M. muris* pretreatment (IAV + NTHi), and this was sustained from day 5 until day 6 (*P* < 0.01) (Fig. 3B).

### *M. muris* challenge induced a short-lived inflammatory local response.

**FIG 4:**
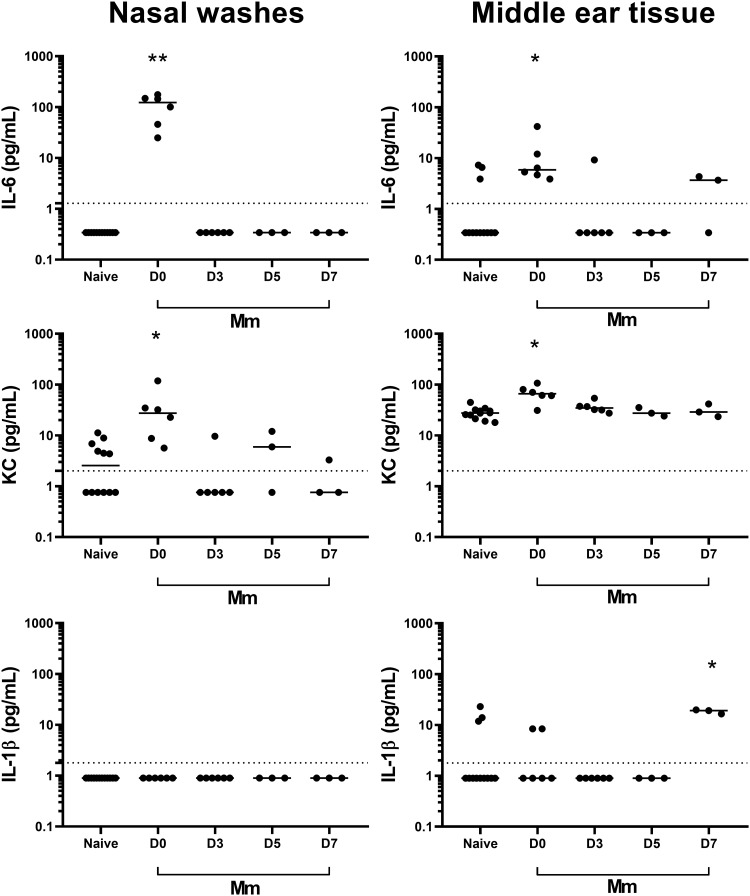
*M. muris* treatment induces a short-lived and local inflammatory response. Inflammatory mediator levels in nasal washes and middle ear tissue homogenate from mice following intranasal challenge with 5 × 10^7^ CFU of *M. muris* on day −1. Specimens were collected on days 0, 3, 5, and 7, which were 1, 4, 6, and 8 days, respectively, after *M. muris* challenge. Each circle represents cytokine levels for an individual mouse. Horizontal bars depict the median analyte titer in picograms per milliliter, and the dashed line represents the assay limit of detection. *, *P* < 0.05; **, *P* < 0.01; ***, *P* < 0.001.

Intranasal challenge with *M. muris* alone induced an inflammatory response in the upper respiratory tract on the day after challenge (day 0, as *M. muris* was administered on day −1), with elevated IL-6 and KC in the nasal washes (*P* > 0.0001 and *P* = 0.0020) and middle ear tissue (*P* = 0.0027 and *P* = 0.0007) compared with those of naive mice (Fig. 4). The IL-6 and KC titers returned to baseline levels by day 3 (4 days after *M. muris* challenge).

**FIG 5:**
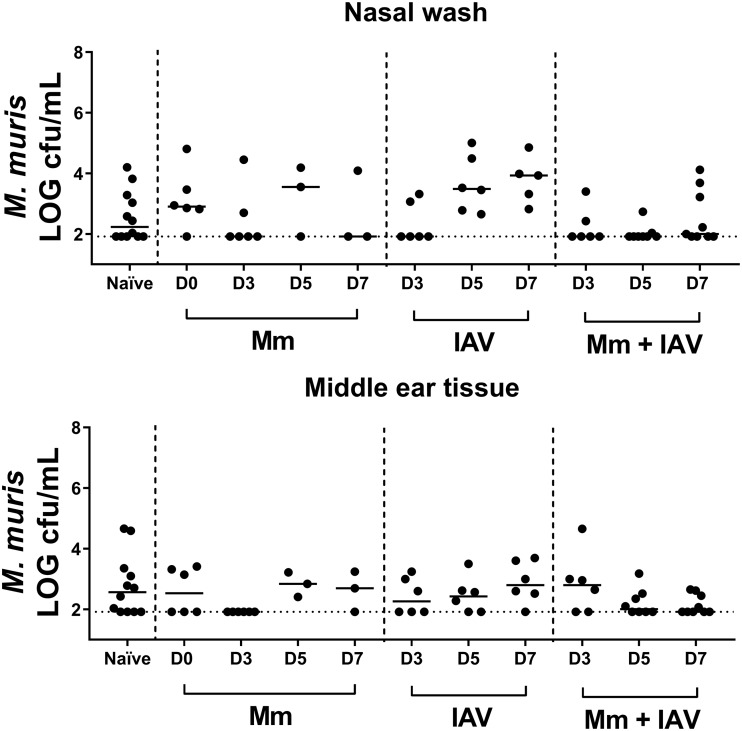
*M. muris*-like counts in nasal washes and middle ear tissue following intranasal challenge with *M. muris* and/or influenza virus. *M. muris*-like log CFU per milliliter counts in nasal washes and middle ear tissue homogenate from naive mice and mice intranasally treated with *M. muris* on day −1 (*M. muris*), influenza A virus on day 0 (IAV), or *M. muris* on day −1 and influenza A virus on day 0 (*M. muris* + IAV). Specimens were collected on days 0 (*M. muris* only), 3, 5, and 7. Each circle represents an individual mouse. Horizontal bars depict the median *M. muris*-like log density, and the dashed line represents the limit of quantification.

To estimate the duration of *M. muris* colonization following *M. muris* treatment (and whether *M. muris* entered the middle ear), all colonies that appeared *M. muris*-like on nonselective chocolate agar plates were counted in nasal washes and middle ear tissue (Fig. 5). It is important to note that asymptomatic colonization with *M. muris* as part of the normal microbiome was present, as indicated by the *M. muris*-like counts in specimens collected from naive mice (Fig. 5). While the median *M. muris*-like counts were higher (but not significantly so) in *M. muris*-treated mice (*M. muris* only group on day 0 and day 5) than in naive mice, *M. muris* counts also increased when mice were given IAV only, indicating that viral infection amplifies the number of resident *M. muris*-like bacteria in the respiratory tract (Fig. 5). *M. muris* challenge did not increase the number of *M. muris*-like colonies recovered from the middle ear, with similar viability counts in middle ear tissue from challenged and naive mice. Viable counts of *M. muris* were not conducted for specimens from mice treated with both *M. muris* and NTHi (*M. muris* + IAV + NTHi), as these species are indistinguishable on nonselective agar plates.

### *M. muris* does not directly inhibit NTHi growth.

*In vitro* coculture tests did not reveal evidence of direct bacterial-bacterial interference between *M. muris* and NTHi.

## DISCUSSION

A noninvasive bacterial therapy to prevent NTHi colonization of the upper respiratory tract is a particularly attractive approach to reduce the burden of otitis media and resulting antibiotic use. Prevention of NTHi otitis media could reduce the global burden of otitis media by approximately 50% (26), resulting in an estimated 350 million fewer cases of acute otitis media episodes per year. This would have a major impact on preventing chronic and recurrent otitis media to improve health and educational outcomes for children throughout the world. In this study, we have demonstrated that a commensal *Pasteurellaceae* species can be applied intranasally to reduce NTHi colonization and delay onset of NTHi otitis media in mice. This builds upon our previous findings that H. haemolyticus can prevent NTHi infection of human respiratory epithelial cells *in vitro* (23).

Nasal probiotic therapies are being investigated to protect against pneumococcal otitis media using commensal alpha-hemolytic streptococci (18, 27–31). In a prospective, randomized, double-blind, placebo-controlled study, 100 Italian children aged 1 to 5 years with histories of recurrent acute otitis media (AOM) were randomized 1:1 to intranasally receive Streptococcus salivarius 24SMB or saline placebo twice daily for 5 days each month for 3 consecutive months (27). Children that were successfully colonized with *S. salivarius* 24SMB were protected from developing otitis media (13.6% versus 42.8%; *P* = 0.03). In our study, only one application of *M. muris* was administered, and the beneficial effects were transient. It is likely that a nasal commensal therapy for NTHi otitis media will also require multiple applications to enhance duration of protection from NTHi colonization and disease. We chose not to decolonize mice with antibiotics prior to this study for removal of endogenous *Pasteurellaceae* species, as the intended application of this bacterial therapy is that it is suitable for all children regardless of the profile of their nasal microbiota.

A human infection study has been conducted with NTHi, where 15 healthy adult volunteers were intranasally challenged with a single ascending dose of NTHi to establish colonization (32). All participants experienced mild clinical symptoms, including rhinorrhea, pharyngitis, and/or headaches. We propose that pretreatment with an intranasal application of a commensal *Pasteurellaceae* species, e.g., H. haemolyticus (the human equivalent of *M. muris*), could prevent development of the NTHi-associated symptoms in this human challenge model. The potential impact of a therapy that prevents NTHi colonization is not limited to otitis media, as prevention of NTHi colonization could also prevent other NTHi-associated diseases such as pneumonia, the major global killer of children under 5 years of age (33), and chronic obstructive pulmonary disease, which is the 3rd most common cause of adult mortality (34).

Inflammation in the middle ear is a major feature of otitis media (35, 36). Our observation that mice pretreated with a single dose of *M. muris* have lower levels of inflammatory mediators in their upper respiratory tract than mice with no *M. muris* pretreatment in the otitis media model further demonstrates the potential use of commensal *Pasteurellaceae* species to prevent NTHi disease. The inflammatory response of the control groups, particularly that seen on initial delivery of *M. muris*, may offer insight into the mechanism behind *M. muris* prevention of NTHi colonization and otitis media. The initial increases in IL-6 and KC on day 0 (24 h after *M. muris* treatment) in both the middle ear and nasal washes of the mice suggest that despite not seeing an increase in the commensal density, an innate immune response was elicited. While inflammation was short-lived, it indicates that immune modulation, rather than physical competition with NTHi, may play a role in microbial interference with *M. muris* protection against NTHi colonization and otitis media. This is further supported by the observation that direct bacterial-bacterial interference was not apparent *in vitro*.

Intranasal influenza virus challenge is essential for ensuring NTHi colonization and driving development of NTHi otitis media in the murine ascension model (37), presumably from NTHi taking advantage of the inflamed respiratory tract of influenza virus-challenged mice. Pretreatment with *M. muris* significantly reduced the nasal inflammatory response to influenza virus, suggesting that *M. muris* alters the host response to viral infection. It is likely that *M. muris* dampening of the innate inflammatory response to influenza virus is associated with reducing the ability of NTHi to colonize and go on to cause otitis media. The improved clinical scores of *M. muris*-pretreated mice are also likely to be a reflection of the lower amount of NTHi that was able to colonize the respiratory tract and, thus, ascend into the middle ear to cause infection and inflammation. A potential explanation for the earlier weight loss response to IAV exposure in *M. muris*-pretreated mice than in mice that were not pretreated with *M. muris* may be the accumulative stress of repeat anesthesia in the *M. muris* + IAV group compared with that of the IAV groups. Of interest is the dampened nasal IL-6 and KC response and middle ear IL-1β response to challenge with NTHi alone, often at or below controls including naive mice. This is a phenomenon that we have previously observed in cell culture experiments where NTHi “flies under the radar” of the host innate immune response and appears to suppress IL-6 and IL-8 production rather than elicit it (23).

In summary, we have demonstrated that intranasal treatment with the commensal *Pasteurellaceae* species *M. muris* can reduce NTHi colonization and prevent development of NTHi otitis media *in vivo*. This work supports further investigation into the potential use of a commensal *Pasteurellaceae* species to prevent NTHi colonization and disease in humans.

## MATERIALS AND METHODS

The sources of the microorganisms used in this study are detailed in Table 1.

**TABLE 1:** Strains of microorganisms used in this study

Species	Strain (reference/source)
Nontypeable Haemophilus influenzae	R2866 (41)
Nontypeable Haemophilus influenzae	R2866 Spec^r^ (this study)
Influenza A virus	A/Memphis/1/71 H3N2 (supplied by Alex Larcombe)
Muribacter muris	TKI (this study)

### Bacterial inoculum.

Standard inoculum of mid-log phase NTHi 2866 Spec^r^ was prepared in 1-ml aliquots and stored as previously described (38), with the exception that 0.1 mg/ml spectinomycin was added to the culture medium. *M. muris* was isolated from the respiratory tract of a mouse in our animal facility by plating a nasal wash onto chocolate agar plates and selecting *Haemophilus*-like colonies. Species identity of a selected isolate (*M. muris* TKI) was confirmed by sequencing of the 16S gene at the Australian Genome Research Facility in Melbourne, Australia. Standard inoculum of *M. muris* was prepared by picking 3 colonies from an overnight chocolate agar plate and seeding into 15 ml culture medium (heart infusion broth supplemented with 44 ml/liter glycerol, 30 mg/liter hemin, and 10 mg/liter NAD). The culture was incubated at 37°C with shaking to mid-log phase (optical density at 600 nm [OD_600_] was between 0.55 and 0.65), then 20% heat-inactivated fetal calf serum was added, and 1-ml single-use aliquots of *M. muris* TKI were prepared and stored in cryovials at –80°C (as for the NTHi inoculum). The number of CFU for each inoculum was determined after at least 24 h storage at –80°C as previously described (38). Viability of the frozen inoculum was assessed over the study period and found to remain stable for both species for at least 12 months.

### Influenza A virus.

For preparation of the influenza virus inoculum, influenza A/Memphis/1/71 H3N2 virus (IAV) was subpassaged through Madin-Darby canine kidney (MDCK) cells (NBL-2, ATCC CCL-34) in Dulbecco’s modified Eagle’s medium (DMEM; Gibco, Sydney, Australia), then harvested from tissue culture supernatant, and viral titers were determined by plaque assay as previously described (39). Viral stocks were stored at −80°C.

### Construction of NTHi R2866 Spec^r^.

The NTHi R2866 Spec^r^ mutant was generated by allelic exchange of pseudogene R2866_1356 with a spectinomycin resistance cassette that was amplified from plasmid pR412 (40) with primers PBpR412_L (5′-GCCGCTCTAGAACTAGTGG-3′) and PBpR412_R (5′-GATACCCCTCGAATTGACGC-3′). The left flanking region of the R2866_1356 gene was amplified with primers R2866_1356_L1 (5′-TCATTTTAGACGGTGCGATG-3′) and R2866_1356_L2 (5′-**CCACTAGTTCTAGAGCGGC**CACGGGAAGCGTTAGAGGTA-3′) from genomic DNA prepared from NTHi strain R2866 (41). The right flanking region of the R2866_1356 gene was also amplified from R2866 genomic DNA using primers R2866_1356_R1 (5′-CACACCCAACCACTTCATCA-3′) and R2866_1356_R2 (5′-**GCGTCAATTCGAGGGGTATC**ACCACAAACTCAACCCAAGC-3′). Primers R2866_1356_L2 and R2866_1356_R2 contain overlapping regions (in bold) with the spectinomycin cassette for the construction of a megaprimer PCR product consisting of the R2866_1356 left flanking region, spectinomycin cassette, and the R2866_1356 right flanking region. The NTHi R2866_1356 gene deletion mutant was obtained by transformation of the megaprimer PCR product using the method of Herriott et al. (42) and selected by plating onto brain heart infusion (BHI) agar plates containing 150 μg/ml of spectinomycin. The gene deletion mutant was validated by PCR with primer sets R2866_1356_L1 + R2866_1356_C (5′-TCGGCAATTGGTACGTTTT-3′) and R2866_1356_L1 + PBMrTn9 (5′-CAATGGTTCAGATACGACGAC-3′) (43), which detect the presence of the R2866_1356 gene or spectinomycin cassette, respectively. Gene deletions were crossed back to the wild-type strain using chromosomal DNA from the mutant strains as the donor during transformation.

### Animals.

All animal experiments were approved by the Telethon Kids Institute Animal Ethics Committee, Perth, Australia (number A302). Female specific-pathogen-free BALB/c mice were obtained from the Animal Resources Centre (Perth, Australia). Experiments were conducted in sets of 12 to 15 mice, ensuring representation from each group at each time point.

### NTHi otitis media model.

Viral coinfection is required for reliable development of NTHi otitis media infection in mice using the ascension model (37). Briefly, 6- to 8-week-old female BALB/c mice were inoculated intranasally with 1 × 10^4.5^ PFU IAV in a volume of 10 μl. At 72 h after IAV challenge, mice were intranasally administered 5 × 10^7^ CFU of NTHi R2866 Spec^r^ in 10 μl of phosphate-buffered saline (PBS). For the groups pretreated with *M. muris*, mice received intranasal inoculation of 5 × 10^7^ CFU of *M. muris* TKI on day −1. Mice were monitored, weighed, and clinically assessed each day. Clinical disease scores were assessed as previously described (44) using a scale ranging from 0 to 20 according to the following criteria: score 0 = normal appearance, healthy, and active; score 1 to 5 = barely ruffled fur, mildly/intermittent hunched appearance, and otherwise healthy; score 6 to 10 = moderately ruffled fur, elevated respiratory rate, hunched appearance with a crab-like gait, intermittent stillness, and reduction of curious behavior; and score 11 to 20 = ruffled fur, labored breathing, hunched appearance with a crab-like gait, and unresponsive to stimuli. Additional control groups included no treatment at all (naive), *M. muris* only, IAV only, NTHi only, and *M. muris* + IAV. Treatment groups, sample size, and experimental time points are detailed in Table 2.

**TABLE 2:** Treatment groups, sample sizes, and number of mice culled at each time point

Group	Timepoint (no. of mice culled)					Total no. in sample
	Day −1	Day 0	Day 3	Day 5	Day 7	
Naive controls		3	3	3	3	12
*M. muris* only		6	6	3	3	18
IAV only			6	6	6	18
NTHi only				6	6	12
*M. muris* + IAV			6	8	9	23
IAV + NTHi				15	21	36
*M. muris* + IAV + NTHi				12	12	24

### Specimen collection and processing.

Nasal washes and middle ear bullae were collected immediately postmortem and stored on ice. The nasal washes were conducted by lavaging the nares with 0.1 ml PBS. Middle ear tissue (combined from both ears of a mouse) was mechanically homogenized in 0.5 ml PBS using hand-held sterile plastic pestles (Interpath) until all tissue was disrupted. Nasal washes and middle ear tissue homogenates were serially diluted in PBS and spotted onto chocolate agar plates with and without an overlay of 200 μl of 10 mg/ml spectinomycin (to select for the NTHi Spec^r^ strain). Remaining middle ear homogenate and nasal washes were centrifuged at 13,000 rpm for 10 min at 4°C to remove cell debris. The supernatants were filtered using 0.2-μm syringe filters and stored in aliquots at –80°C for subsequent measurement of inflammatory mediators.

### Measurement of inflammatory mediators in nasal washes and middle ear tissue.

Stored supernatants from nasal washes and middle ear tissue homogenates were tested using a Bio-Rad express assay 5-plex murine cytokine/chemokine magnetic bioplex kit to measure IFN-γ, IL-1β, IL-6, KC, and IL-10 on the BioPlex 2000 (Bio-Rad) according to the manufacturer’s instructions. Where cytokine titers were below the limit of detection (LOD), half of the value of the lowest standard was assigned to permit statistical analysis. The LOD of each cytokine was as follows: IFN-γ = 0.94 pg/ml, IL-1β = 1.79 pg/ml, IL-6 = 0.68 pg/ml, KC = 1.51 pg/ml, and IL-10 = 4.62 pg/ml.

### Assessment of bacterial interference.

The following three *in vitro* methods were used to assess bacterial-bacterial interference between *M. muris* and NTHi using previously described methods: direct coculture in broth (45), spot agar test (45), and a well-diffusion assay (24).

### Statistical analysis.

Mann-Whitney U tests were applied to nonparametric data (bacterial counts, CFU/ml; cytokine levels, pg/ml), with a *P* value of <0.05 considered significant. Fisher’s exact testing was used for categorical analyses (development of otitis media). Mean clinical scores and percent weight loss were compared by Student's *t* test.

## Supplementary Material

Supplemental file 1
