# Supplementary material for: Nasal Delivery of a Commensal Pasteurellaceae Species Inhibits Nontypeable Haemophilus influenzae Colonization and Delays Onset of Otitis Media in Mice
Source: Infect Immun. 2020 Mar 23;88(4):e00685-19. doi: 10.1128/IAI.00685-19 (PMC7093147; doi:10.1128/IAI.00685-19)
Supplement: Supplemental file 1 [file IAI.00685-19-s0001.pdf]

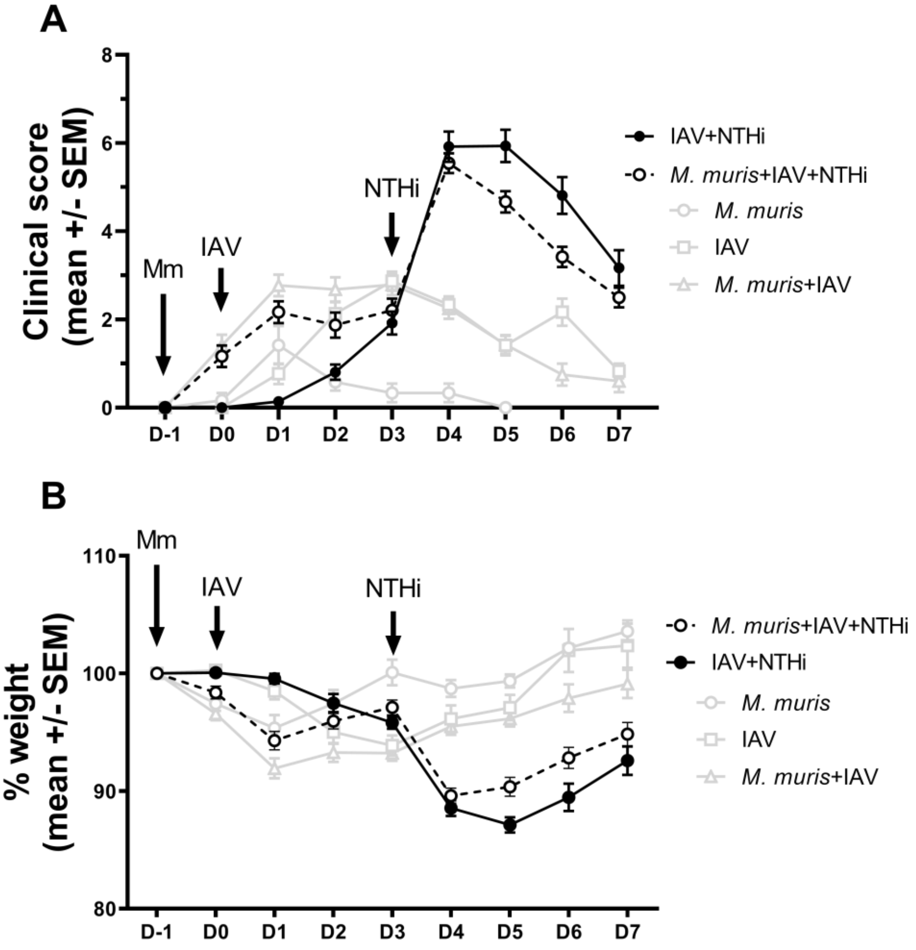

Figure S.1. Pre-treatment of mice with *M. muris* had minimal impact on clinical score and weight loss.

Mean clinical score (A) and weight loss (B) for all treatment groups of mice. Values are presented as the mean +/- standard error of the mean. D = Days post challenge, with influenza A challenge (IAV) as the reference point. NTHi was administered on Day 3 and mice were monitored to Day 7 (*M. muris* only to Day 4); * p<0.01 when compared between Mm+IAV+NTHi and IAV+NTHi. Black lines with filled black circles represent mice that did not receive *M. muris* (Mm) pre-treatment prior to IAV+NTHi; black dashed lines with open white circles represent mice that were pre-treated intranasally with *M. muris* on Day –1 prior to IAV+NTHi (Mm+IAV+NTHi). Grey lines and open shapes represent control groups of mice that were treated with either *M. muris* only, IAV only or *M. muris* + IAV.
